# Supplementary material for: Comparison of Gene Expression Changes in Three Wheat Varieties with Different Susceptibilities to Heat Stress Using RNA-Seq Analysis
Source: Int J Mol Sci. 2022 Sep 14;23(18):10734. doi: 10.3390/ijms231810734 (PMC9505106; doi:10.3390/ijms231810734)
Supplement: Supplementary file 1 [file ijms-23-10734-s001.zip › supplementary data-vf.pdf]

Supplementary data

Comparison of gene expression changes in three wheat varieties with different susceptibility under heat stress using RNA-seq expression analysis

Myoung Hui Lee, Kyeong-Min Kim, Wan-Gyu Sang, Chon-Sik Kang and Changhyun Choi*

National Institute of Crop Science, RDA, Wanju-gun, 55365

*** Corresponding author:** Changhyun Choi
Email: chchhy@korea.kr

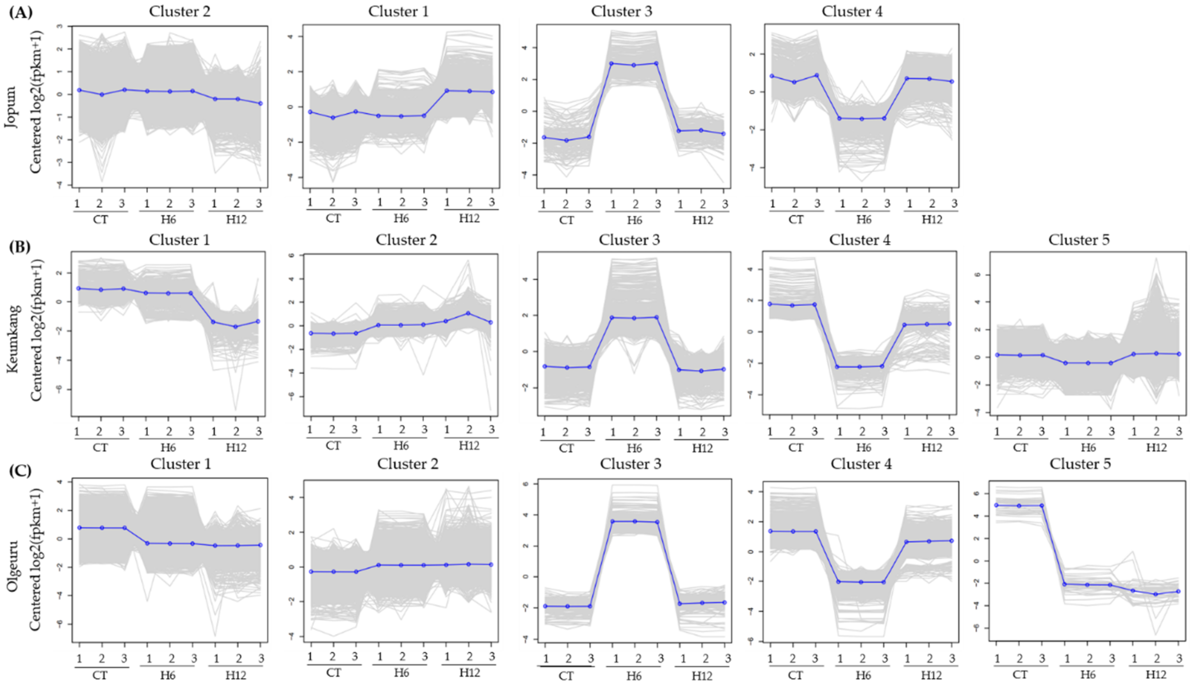

(D)

cultivar	Cluster 1	Ccluster 2	Cluster 3	Cluster 4	Cluster 5	SUM
Jopum	4,376 (49.94%)	3,286 (37.49%)	177 (2.043%)	192 (2.13%)	-	8,763
Keumkang	403 (4.46%)	659 (7.30%)	666 (7.38%)	665 (7.37%)	7,108 (78.73%)	9,028
Olgeuru	4,410 (27.11%)	10,730 (65.96%)	172 (1.057%)	913 (5.61%)	42 (0.258%)	16,267

Figure S1. Clustering results of time-course data from RNA-seq analysis. (A-C) Change in expression pattern of the induced genes in heat-treated groups of Jopum (A), Keumkang (B), and Olgeuru (C). (D) The numbers of DEGs found after heat treatment.

Table S1. Summary of RNA-seq samples and their mapping rate.

Sample		Run format	Max Read Length	Total Reads	Mapped Reads	Mapping Rates (%)
Jopum	0 h-1	101x2	101	33,210,049	27,173,835	81.824
	0 h-2	101x2	101	39,231,412	19,526,044	49.771
	0 h-3	101x2	101	30,790,153	25,423,892	82.572
	6 h-1	101x2	101	27,196,243	22,778,653	83.757
	6 h-2	101x2	101	29,668,213	24,757,249	83.447
	6 h- 3	101x2	101	25,605,162	21,494,302	83.945
	12 h-1	101x2	101	33,491,713	28,025,308	83.678
	12 h-2	101x2	101	33,919,563	28,460,345	83.905
	12 h-3	101x2	101	47,612,081	25,754,508	54.092
Keumkang	0 h-1	101x2	101	35,827,539	29,802,857	83.184
	0 h-2	101x2	101	27,388,739	22,591,607	82.485
	0 h-3	101x2	101	33,343,846	27,748,295	83.219
	6 h-1	101x2	101	26,752,450	21,697,541	81.105
	6 h-2	101x2	101	25,536,743	20,587,733	80.620
	6 h-3	101x2	101	25,335,795	20,450,575	80.758
	12 h-1	101x2	101	27,321,991	22,538,151	82.491
	12 h-2	101x2	101	27,875,334	20,235,153	72.592
	12 h-3	101x2	101	29,954,936	24,683,257	82.401
Olgeuru	0 h-1	101x2	101	36,700,371	31,215,783	85.056
	0 h-2	101x2	101	38,706,882	32,912,414	85.030
	0 h-3	101x2	101	31,887,759	27,462,641	86.123
	6 h-1	101x2	101	32,945,568	27,134,908	82.363
	6 h-2	101x2	101	29,720,915	24,529,545	82.533
	6 h-3	101x2	101	27,569,221	22,631,522	82.090
	12 h-1	101x2	101	26,660,838	21,041,885	78.924
	12 h-2	101x2	101	27,719,742	22,455,168	81.008
	12 h-3	101x2	101	27,752,632	22,547,462	81.244

Table S2. The numbers of significant DEGs under different heat treatment times.

Sample		Up-regulation log2FC>1 & padj <0.05	Down-Regulation log2FC>1 & padj <0.05	total
Jopum	0 h vs 6 h	7,022 (13.78)	4,729 (9.28%)	50,945
	0 h vs 12 h	3,368 (6.78%)	5,482 (10.97%)	49,976
	6 h vs 12 h	5,412 (10.66%)	9,299 (18.31%)	50,774
Keumkang	0 h vs 6 h	6,640 (12.73%)	4,319 (8.28%)	52,167
	0 h vs 12 h	3,621 (7.2%)	4.325 (8.6%)	50,260
	6 h vs 12 h	5,728 (10.88%)	8,477 (16.09%)	52,671
Olgeuru	0 h vs 6 h	11,302 (21.01%)	11,593 (21.55%)	53,801
	0 h vs 12 h	6,201 (21.31%)	6,859 (13.62%)	50,362
	6 h vs 12 h	9,102 (16.22%)	10,306 (18.36%)	56,120

Table S3. Significantly enriched biological processes (BP) for differentially expressed genes (DEG) identified in Jopum, Keumkang, and Olgeuru**.** FDR, false discovery rate; FC, fold change, CT, non-heat stress conditions; H6, heat-stress conditions at 35°C for 6 h; H12; heat-stress conditions at 35°C for 6 h.

Cultivar (Criteria)	Comparisons	GO number				Total genes			
		BP	CC	MF	Sum	BP	CC	MF	Sum
Jopum (FDR = 0.05, ∣FC∣>2**)**	CT vs. H6	133	30	124	291	2499	4594	4469	4050
	CT vs. H12	151	28	136	315	2106	1506	3842	7233
	H6 vs, H12	213	49	185	447	3580	1995	4087	10514
Keumkang (FDR = 0.05, ∣FC∣>2)	CT vs. H6	150	34	136	320	2781	2346	2636	7763
	CT vs. H12	107	35	98	230	1443	1059	2322	4824
	H6 vs, H12	201	37	65	403	3332	3042	3667	10041
Olgeuru (FDR = 0.05, ∣FC∣>2)	CT vs. H6	209	43	159	412	4218	6160	8688	19066
	CT vs. H12	221	48	188	458	2701	2696	4896	10287
	H6 vs, H12	148	35	121	304	3741	8817	7805	20357

Table S4. Summary of enriched biological processes (BP) for differentially expressed genes (DEG) in each comparison. FDR, false discovery rate; FC, fold change, CT, non-heat stress conditions; H6, heat-stress conditions at 35°C for 6 h; H12; heat-stress conditions at 35°C for 12 h.

Cultivar (Criteria)	Comparisons	GO term					Total genes	
		three comparisons	two comparisons	single comparison	BP	Sum	BP	Sum
Jopum (FDR = 0.05, ∣FC∣>2)	CT vs. H6			34	133	291	2499	4050
	CT vs. H12	38	125	33	151	315	2016	7233
	H6 vs, H12			64	213	447	3580	10514
Keumkang (FDR = 0.05, ∣FC∣>2)	CT vs. H6			45	150	320	2781	7763
	CT vs. H12	34	98	30	107	230	1443	4824
	H6 vs, H12			78	201	403	3332	10041
Olgeuru (FDR = 0.05, ∣FC∣>2)	CT vs. H6			50	209	412	4218	19066
	CT vs. H12	39	76	97	221	458	2701	10287
	H6 vs, H12			42	148	304	3741	20357

Table S5. Identification of ROS-scavenging genes in leaves of wheat seedlings inn response to heat treatment.

Transcript ID	Gene description	Log2 Readcount ratio						p-value					
		Jopum		Keumkang		Olgeuru		Jopum		Keumkang		Olgeuru	
		6h/0h	12h/0h	6h/0h	12h/0h	6h/0h	12h/0h	6h/0h	12h/0h	6h/0h	12h/0h	6h/0h	12h/0h
TraesCS5A02G498000.1	Catalase	1.515	0.230	2.144	1.009	3.529	1.427	1.09826E-10	0.518239999	8.2112E-302	3.12915E-06	0	4.14311E-90
TraesCS4D02G322700.1	Catalase	1.021	0.336	1.768	1.142	3.108	1.295	1.01425E-05	0.336345911	1.5335E-198	1.14049E-07	0	1.17822E-42
TraesCS4B02G325800.1	Catalase	0.987	0.116	1.732	0.641	2.967	1.080	5.68015E-05	0.75232867	9.8405E-206	0.003529013	0	2.51844E-47
TraesCSU02G105300.1	Catalase	-0.456	0.928	-1.047	0.585	-1.922	-1.370	0.006674	0.000125152	1.2775E-147	0.005003984	2.8189E-288	1.38732E-58
TraesCS6D02G048300.1	Catalase	5.6831	2.838	-0.733	-	-6.990	-	0.000176261	0.18174179	0.726165336	-	0.005783219	-
TraesCS6D02G048300.3	Catalase	4.2796	4.060	0.551	10.408	-2.008	0.070	4.9535E-105	1.63261E-42	0.001472695	2.5465E-42	4.928E-269	0.448588599
TraesCS7A02G549800.1	Catalase	0.2216	0.726	-0.940	-0.017	-3.196	-1.970	0.173593126	0.002642342	2.87352E-12	0.959790932	8.00098E-60	2.89441E-20
TraesCS7A02G549800.2	Catalase	-0.052	1.205	-0.988	1.023	-1.934	-1.398	0.757737434	0.000258563	5.56744E-80	0.046954933	2.57779E-51	1.22211E-22
TraesCS6A02G041700.1	Catalase	2.917	5.273	1.878	10.793	-1.9007	4.616	1.62945E-49	4.28987E-55	2.52143E-31	2.59238E-47	2.61058E-33	6.80439E-06
TraesCS2B02G604800.1	Glutathione peroxidase	1.954	2.203	4.584	3.534	6.888	5.634	7.71115E-51	1.64264E-27	0	1.50116E-38	0	0
TraesCS2D02G598000.1	Glutathione peroxidase	1.109	2.655	3.661	4.657	3.440	5.778	1.01345E-08	2.95409E-13	0	3.18178E-55	0	2.285E-283
TraesCS2A02G582100.1	Glutathione peroxidase	0.682	1.341	2.638	2.538	4.855	5.070	5.5058E-06	1.43908E-08	2.3396E-129	6.20094E-28	0	1.4098E-302
TraesCS3D02G299900.1	Glutathione S-transferase	0.396	0.279	-0.008	-0.064	-2.105	-0.049	0.001194516	0.148713439	0.913021537	0.850988083	1.7012E-254	0.765058786
TraesCS2A02G437700.1	ascorbate peroxidase 4	-0.788	-1.392	1.551	-0.466	2.553	1.671	2.4038E-07	6.37127E-08	0	0.067165736	0	2.1864E-134
TraesCS2D02G434500.1	ascorbate peroxidase 4	-0.370	-1.555	1.582	-0.283	3.068	1.909	0.006187547	1.26665E-12	0	0.187574435	0	8.3218E-147
TraesCS2B02G457600.1	ascorbate peroxidase 4	-1.758	-2.490	0.979	-0.579	2.860	3.141	4.47152E-42	3.06336E-34	1.7159E-138	0.017210785	0	1.0026E-275
TraesCS6A02G412900.1	L-ascorbate peroxidase 8	-2.169	-2.287	0.349	-0.410	2.742	2.059	7.97295E-32	3.60046E-15	4.32442E-42	0.043727863	0	1.04044E-58
TraesCS7D02G249200.1	L-ascorbate peroxidase 6	1.301	-1.887	1.807	-1.380	2.824	-0.223	1.38321E-12	6.65966E-20	7.22551E-55	5.44284E-10	1.0957E-143	0.017069993
TraesCS6A02G383800.2	Glutathione reductase	0.563	0.067	-	0.244	2.074	-0.009	0.0111533	0.825169893	-	0.304342929	7.3105E-168	0.915503673
TraesCS6D02G303900.1	Peroxidase	-	-	-	-	7.941	7.456	-	-	-	-	1.44324E-28	5.3951E-23

TraesCS2D02G107600.1	Peroxidase	3.964	4.9154	-0.756	6.625	-0.914	4.667	4.876E-105	1.92124E-67	1.52388E-63	1.52809E-88	1.22415E-59	0
TraesCS1D02G096400.1	Peroxidase	-3.023	-4.430	-3.184	-0.667	0.877	3.915	3.96446E-10	3.01885E-22	0	0.03961762	7.42685E-07	2.33331E-32
TraesCS7D02G347300.1	Peroxidase	0.203	0.401	-1.894	-0.553	-2.020	3.283	0.692158653	0.388225335	3.63176E-11	0.207659083	8.1389E-41	4.6962E-08
TraesCS1A02G077700.2	Peroxidase	-1.195	-2.213	0.692	-0.685	3.212	3.086	0.039306587	0.000117575	0.000636941	0.021964777	2.9873E-20	1.009E-46
TraesCS2B02G125800.1	Peroxidase	2.075	1.717	1.115	0.535	1.181	2.941	3.88813E-13	3.62543E-08	8.19528E-14	0.037564558	4.42014E-35	1.20347E-68
TraesCS2D02G108500.1	Peroxidase	0.903	1.511	-0.450	0.913	-0.213	2.911	0.015417199	0.001192291	0.009480604	0.018567345	0.089578084	2.60409E-26
TraesCS6B02G063900.1	Peroxidase	-0.510	3.940	-2.23	3.370	-0.136	2.590	0.000370178	1.53183E-56	2.967E-211	1.70414E-33	0.029017307	1.6979E-116

Table S6. Heat stress transcription factor (*HSF*) genes in response to heat treatment in leaves of wheat seedlings.

Gene ID	Gene description	Log2 Readcount ratio						P-value					
		Jopum		Keumkang		Olgeuru		Jopum		Keumkang		Olgeuru	
		6h/0h	12h/0h	6h/0h	12h/0h	6h/0h	12h/0h	6h/0h	12h/0h	6h/0h	12h/0h	6h/0h	12h/0h
TraesCS5B02G236400.1	HSF Spl7	-2.089	-0.424	-2.8801	0.623	-3.285	-0.937	9.17864E-20	0.239287909	4.60793E-40	0.228961664	1.24094E-48	0.017946097
TraesCS1A02G350400.2	HSF Spl7	2.373	-0.210	-0.310	1.869	-2.756	0.144	2.39426E-07	0.592999512	0.459448909	0.041513191	2.06658E-41	0.73715695
TraesCS2D02G399000.1	HSF Spl7	-3.632	-	-2.319	-0.140	-4.565	-0.561	1.89664E-26	-	5.04347E-17	0.72952597	5.6735E-129	0.248073048
TraesCS5A02G237900.1	HSF Spl7	-2.013	-0.557	-3.355	0.934	-4.851	-1.613	9.13108E-27	0.04046389	3.5137E-122	0.019385008	2.3845E-110	0.00093471
TraesCS2A02G401600.1	HSF Spl7	-8.456	-	-5.623	-	-6.685	-	7.36531E-45	-	1.54152E-29	-	3.21918E-64	-
TraesCS5D02G244800.1	HSF Spl7	-0.760	0.016	-2.475	1.204	-3.403	-0.925	2.50832E-07	0.982548077	2.36102E-64	0.001412233	6.1331E-127	0.000370058
TraesCS5A02G314700.1	HSFB-2c	-2.921	-0.402	-3.019	0.053	-2.573	-0.319	8.77843E-89	0.037574092	1.1208E-222	0.89455597	5.2386E-247	0.047771597
TraesCS5D02G321000.1	HSFB-2c	-2.906	-0.132	-2.887	-0.087	-2.582	-0.288	1.50731E-61	0.564767513	2.1808E-112	0.823068457	1.2633E-108	0.211876818
TraesCS5B02G315600.2	HSFB-2c	-3.144	-0.577	-2.982	-0.122	-2.030	0.497	1.55493E-51	0.035796634	8.35999E-66	0.690796924	3.32383E-56	0.06659075
TraesCS5A02G437900.1	HSF2C	-2.596	-1.204	-2.341	-0.661	-1.443	-0.493	2.4788E-71	9.89798E-09	0	0.002329009	0.002329009	2.07161E-06
TraesCS5D02G445100.1	HSF2C	-3.131	-1.415	-2.577	-1.018	-2.306	-0.814	5.32626E-41	3.80778E-05	1.19E-132	4.06433E-05	3.5926E-131	0.000137088
TraesCS5B02G440700.1	HSF2C	-2.725	1.130	-2.176	-1.202	-1.162	-0.366	0.000183771	0.113189683	0.008084159	0.201219084	5.52834E-07	0.161992888
TraesCS5D02G445100.2	HSF2C	-0.343	-0.287	-0.730	-0.352	-1.451	-1.051	0.142039946	0.30637625	0.039171487	0.715735162	3.1582E-15	5.24287E-05
TraesCS6A02G098800.1	HSF2C	-	-	-1.200	0.218	-1.318	-0.253	-	-	1.76197E-05	0.606389845	2.39763E-07	0.476062422
TraesCS6D02G083800.1	HSF2C	-1.109	-	-	-	-2.185	-	0.065565287	-	-	-	6.11877E-06	-
TraesCS1A02G375600.2	HSFA-2c	-5.789	-0.470	-5.366	0.165	-5.207	0.363	4.85342E-13	0.537114843	0	0.700499355	0	0.151390434
TraesCS1A02G375600.1	HSFA-2c	-2.266	-	-9.589	-	-10.069	-	0.240478472	-	2.68907E-38	-	1.79261E-34	-
TraesCS7A02G270100.1	HSFB-2b	-5.029	-	-5.702	-	-12.389	-	3.96839E-65	-	8.2653E-124	-	4.1739E-246	-
TraesCS3A02G280800.1	HSFC1b	-2.518	-2.234	-2.420	-3.278	-2.700	-3.739	4.33313E-07	0.000401551	1.36871E-12	7.49826E-10	3.92682E-13	2.20148E-26

Table S7. Heat shock protein (HSP) genes in response to heat treatment in leaves of wheat seedlings.

Gene ID	Gene description	Log2 Readcount ratio						P-value					
		Jopum		Keumkang		Olgeuru		Jopum		Keumkang		Olgeuru	
		6h/0h	12h/0h	6h/0h	12h/0h	6h/0h	12h/0h	6h/0h	12h/0h	6h/0h	12h/0h	6h/0h	12h/0h
TraesCS3D02G273600.1	HSP101c	-10.645	-	-11.866	-	-10.898	-	4.4832E-205	-	0	-	0	-
TraesCS7D02G241100.1	HSP90	-4.565	-0.729	-5.102	-0.847	-5.040	-0.605	1.81303E-62	0.002125882	0	8.00993E-05	0	1.77791E-10
TraesCS7B02G149200.1	HSP90	-4.654	-1.055	-5.156	-0.960	-4.886	-0.483	3.05188E-60	4.83448E-05	0	5.35586E-07	0	9.64423E-13
TraesCS5D02G268000.1	HSP90	-2.031	0.316	-2.426	0.0717	-2.475	-0.410	2.84732E-21	0.220449933	0	0.706500181	0	3.7851E-07
TraesCS5D02G113700.1	HSP90	-1.310	-0.574	-0.783	1.086	-2.177	-1.832	1.11936E-08	0.113158273	7.80499E-07	0.178617907	1.184E-17	1.08851E-14
TraesCS5D02G113700.2	HSP90-6	-0.578	-0.317	-1.479	-0.472	-2.950	-1.786	0.000930979	0.141862113	7.7516E-147	0.021907742	2.1662E-270	1.10001E-24
TraesCS2B02G047400.1	HSP90.1-B1	-8.216	-	-8.142	-	-10.744	-	2.1806E-154	-	0	-	0	-
TraesCS5A02G101900.1	HSP90	0.1184	-0.655	-1.043	-0.504	-1.990	-2.031	0.305977359	0.000333077	2.5466E-103	0.020989478	1.48457E-18	1.41787E-17
TraesCS5B02G258900.1	HSP90	1.4000	5.106	0.170	0.750	2.402	-1.846	0.596755321	0.083342196	0.936836674	0.782371907	0.266503822	0.267567183
TraesCS5B02G258900.2	HSP90	-2.084	0.030	-2.539	-0.295	-2.438	-0.355	1.07012E-22	0.909840818	0	0.147468353	3.6476E-292	0.062549603
TraesCS5B02G258900.3	HSP90	-3.558	-	-3.999	-	-8.384	-	0.001459393	-	0.000603308	-	2.07932E-14	-
TraesCS5B02G258900.4	HSP90	-2.866	9.474	-5.207	-	-	-	0.001336291	1.34339E-05	7.867E-169	-	-	-
TraesCS5A02G260600.1	HSP90	-	-	-1.614	-0.419	-2.054	-1.320	-	-	7.10775E-07	0.196901568	1.34587E-05	0.005349018
TraesCS5A02G260600.4	HSP90	-	-	-2.841	6.757	0.140	-	-	-	0.038848336	0.013561261	0.966770638	-
TraesCS5B02G106300.1	HSP90	-2.572	-0.931	-2.862	-0.727	-1.718	-0.331	1.62778E-50	6.93586E-06	0.007206108	0.007206108	9.2494E-106	0.00055282
TraesCS4A02G066100.1	HSP70	-9.151	-	-5.601	-	-4.761	-	3.15755E-20	-	6.12757E-11	-	1.2925E-08	-
TraesCS4B02G243400.1	HSP70	-4.764	-	-5.806	-	-3.824	-	2.40133E-51	-	5.1133E-120	-	2.32702E-28	-
TraesCS4D02G243000.1	HSP70	-6.440	-	-7.751	-	-8.452	-	1.97857E-28	-	6.12221E-37	-	0.248233498	-
TraesCS1D02G284000.2	HSP70	-5.943	-1.041	-6.062	-0.776	-6.247	-0.623	7.0119E-104	1.20431E-06	0	0.00115552	0	8.00068E-06
TraesCS6D02G169100.1	HSP26.5	-7.653	-	-6.689	-	-9.868	-	3.9728E-22	-	1.5088E-41	-	5.33443E-39	-
TraesCS1D02G319400.1	HSP21.7	1.416	0.012	1.959	0.139	2.182	0.260	2.62781E-14	1	2.61687E-22	0.729769752	9.09783E-32	0.127122572
TraesCS1B02G331900.1	HSP21.7	1.298	-0.016	1.411	0.234	2.265	0.575	2.16726E-08	0.919365874	1.63995E-21	0.533096439	5.44898E-05	-
TraesCS1A02G319600.1	HSP21.7	1.465	0.137	1.330	0.488	2.234	0.136	5.76355E-09	0.632384099	1.54859E-12	0.178200029	4.88932E-42	0.370869575
TraesCS2B02G247300.1	HSP15.4	1.168	-0.023	2.537	0.272	0.655	0.589	5.24598E-06	0.975561639	1.02113E-38	0.349949045	0.000909567	0.008420708
TraesCS7B02G109100.1	HSP15.7	-1.867	-0.416	-2.237	-0.989	-2.594	-1.446	1.92782E-26	0.10071447	2.54107E-42	0.046148226	6.94878E-51	1.29956E-05
TraesCS7D02G205400.1	HSP15.7	-4.406	-1.672	-5.043	-1.998	-4.258	-1.319	1.08321E-56	4.40144E-05	7.9191E-202	0.001152519	1.478E-119	0.002248841
TraesCS7A02G202200.1	HSP15.7	-6.355	-	-8.403	-	-11.639	-	1.03715E-13	-	8.45E-15	-	6.08E-128	-
TraesCS3A02G033900.1	SHSP	-7.831	-3.610	-7.286	-2.990	-10.310	-	3.37498E-98	1.28428E-05	3.6312E-269	0.000590717	1.56838E-49	-
TraesCS4B02G225400.1	SHSP	-6.355	-	-8.403	-	-11.639		1.03715E-13	-	8.4511E-15	-	6.0822E-128	-
TraesCS7A02G177500.1	HSP23.6	-3.958	0.002	-4.203	-0.454	-5.446	-0.002	3.62521E-48	-	0.000426849	0.787623596	1.9312E-214	1
TraesCS7A02G177700.1	HSP23.6	-11.388	-	-4.843	-2.096	-11.206	-	1.05889E-39	-	0.015125423	0.728150661	3.06417E-41	-

TraesCS3D02G114900.1	HSP17.8	-8.184	-	-9.710	-	-11.962	-	2.6903E-108	-	0	-	5.487E-208	-
TraesCS6D02G322300.1	HSP17.4	-10.269	-4.050	-9.744	-4.250	-9.850	-	7.7702E-194	8.96389E-10	0	2.59428E-06	0	-
TraesCS4D02G226000.1	HSP26	-9.058	-4.583	-9.341		-7.353	.	6.7763E-124	7.39773E-07	0	-	3.05454E-94	-

Table S8. Primers used in this study.

Gene name	Primer name	direction (5'-3′)	transcript ID	Accession no.
HSP101c	HSP101c-F<br>HSP101c-R	AAGCTCGGCTAGTTGATGTG<br>CCTCTCTTTCTCCTTGCGATAC	TraesCS3D02G273600.1	AF174433.1
Hsp90.1-B1	hsp90.1b1-F<br>hsp90.1b1-R	CTCAAGGTCATCCGCAAGAA<br>TGGAGAAGGCCTCGTAGAA	TraesCS2B02G47400.1	GQ240782.1
Hsp90-6	Hsp90-6-F<br>HSFA-2c-R	GGACTGGGAGCTTACAAATGA<br>CTCCTGCCTTAGCTTCACTAC	TraesCS5D02G113700.1	XP_044395783.1
HSP 70-8-like	Hsp70-8-F<br>Hsp70-8-R	TGGGAAGTGGCATTGAGAAA<br>CAACCTGCGAGTGCTCTTAT	TraesCS4A02G066100.1	XP_044376084.1
TaHSP23.9	TaHSP23.9-F<br>TaHSP23.9-R	GAGACCTCCGACTCCCAC<br>CGCCCTTCCGTTCTTCCA		A0A3B6B0K8
HSFA-2c-LIKE	HSFA-2c-F<br>HSFA-2c-R	GAGGTTGGTGAGTTTGGATTTGA<br>CTCCTGCCTTAGCTTCACTAC	TraesCS1A02g375600.1	XP_037425119.1
HSFB-2b-like	HSFB-2b-F<br>Hsp70-8-R	CAGCTCAACACCTACGGATTTA<br>CAACCTGCGAGTGCTCTTAT	TraesCS7A02G270100.1	XP_044433701.1
TaHSF3	TaHSF3-F<br>TaHSF3-R	CCAACTCACACCTACGGA<br>CACCTTTCGCCGATGTAT		TaeJQ771755
TaHsfA6f	TaHsfA6f-F<br>TaHSP23.9-R	ACGACTTCTGGGAGGAGCTG<br>CGCCCTTCCGTTCTTCCA		KJ774108
CAT-5A	CAT-5A-F<br>CAT-5A-R	AAGACCCACATCCAGGAGAA<br>TCGTCGAAGAGGAAGGTGAA	TraesCS5A02G498000.1	XP_044383893.1
CAT-6D	CAT-6D-F<br>CAT-6D-R	TCAAGCCCAACCCAAAGT<br>GTCGTCGAAGAGGAAGAAGAAG	TraesCS6D02G048300	XP_044407354.1
CAT-7A	CAT-7A-F<br>CAT-7A-R	CGGTTATTGTCCGGTTCTCTAC<br>GTCAAAGTTACCCTCTCTGGTG	TraesCS7A02G549800	XP_044425442.1
GPX-2A	GPX-2A-F<br>GPX-2A-R	GCAGGAACCAGATAGTGATGAG<br>GCAGCATTGTTGCCATTCA	TraesCS2A02G582100.1	*GPX-2A*
GPX-2A	GPX-2A-F	GCAGGAACCAGATAGTGATGAG	TraesCS2A02G582100.1	XP_044327006.1

	GPX-2A-R	GCAGCATTGTTGCCATTCA		
GST-3D	GST-3D-F	CCATTCTCTTCGAGTGCCTTATC	TraesCS3D02G299900.1	CAD29480.1
	GST-3D-R	GCCAGCACGTTCTTGATCTTA		
APX-2B	APX-2B-F	CGCTCCGAGTTCCAATCAA	TraesCS2B02G457600.1	XP_044458835.1
	APX-2B-R	TTCAGTGCCAAGGTCAAGAG		
APX-6A	APX-6A-F	GATGAAATATGGGCGGGTAGAT	TraesCS6A02G412900.1	XP_044405307.1
	APX-6A-R	CCTAAGGTGTTCAGCAGGTAAA		
GR-6A	GR-6A-F	GAATCCTTGGTGGTTCTGGT	TraesCS6A02G383800.2	XP_044421617.1
	GR-6A-R	GTCGTGTGCCTTTGCTTTG		
POX-1A	POX-1A-F	TCCCTGACTCCACCTTCAC	TraesCS1A02G077700.2	XP_044387153.1
	POX-1A-R	ACAGGATCACCAGCTCCTC		
POX-1D	POX-1D-F	GGCTTCACGTCCAACTTCT	TraesCS1D02G096400.1	XP_ 044389460.1
	POX-1D-R	GTGGCGTTTCTGAACTTCTCT		
POX-6~~B~~	POX-6D-F	TCCTCCACCTTCGACATTCT	TraesCS6B02G303900.1	XP_044414283.1
	POX-6D-R	AGAGGTTGCAGTTGCCTATTC		
